# Impact of Digital Interventions on the Treatment Burden of Patients With Chronic Conditions: Systematic Review

**DOI:** 10.2196/66874

**Published:** 2025-11-21

**Authors:** Manria Polus, Pantea Keikhosrokiani, Olli Korhonen, Woubshet Behutiye, Minna Isomursu

**Affiliations:** 1 Faculty of Information Technology and Electrical Engineering University of Oulu Oulu Finland; 2 Faculty of Medicine University of Oulu Oulu Finland

**Keywords:** chronic illness, treatment burden, eHealth, mHealth, digital health, systematic review, chronic condition, treatment adherence, telehealth, informational resources, self-management, digital intervention

## Abstract

**Background:**

Digital interventions can provide cost-effective, quality health care for patients with chronic conditions. Patients with chronic conditions often are burdened by a substantial load of adhering to a treatment regimen and suffer from impacts on their function and well-being. This treatment burden has consequences for treatment adherence and disease outcomes. Digital interventions have the potential to alleviate the burden, but they also may cause new challenges and an increased workload for the patient. Previous reviews have examined digital interventions or treatment burden separately, but there is a lack of systematic reviews on the intersection of digital interventions, treatment burden, and chronic conditions.

**Objective:**

This systematic review aimed to evaluate the evidence of how digital interventions impact the treatment burden experienced by people with chronic conditions, and to assess the quality of this evidence.

**Methods:**

We searched databases PubMed, Scopus, Web of Science, ACM, PubMed Central, and CINAHL for articles published between January 1, 2013, and June 17, 2025. We included studies that had key topics related to chronic conditions, treatment burden, and digital interventions. A total of 2 reviewers independently screened the articles in 2 stages, extracted data on study design, participant characteristics, intervention type, and treatment burden outcomes from included articles, and assessed their quality using the Critical Appraisal tools from the Joanna Briggs Institute. A convergent integrated approach was used for data synthesis and integration, where quantitative data were converted into qualitative data, and the qualitative and quantitative evidence were analyzed and categorized together.

**Results:**

We included 46 relevant studies in total. We categorized the interventions into 4 types: Telehealth, informational resources, self-management tools, and facilitated tools. The results of this study indicate that digital interventions mostly support patients with chronic conditions with their treatment burden, with minor concerns of increasing treatment burden. The main benefits are support with self-management, informational support, and easier ways to contact health care professionals. The main concerns were accessibility issues, time-consuming tools, and causing fear and anxiety.

**Conclusions:**

Our findings demonstrate how treatment burden is a relevant concept for future digital health care research and practice. Digital interventions can help patients with their treatment burden by supporting self-management, improving access to health care, improving patients’ experience, and addressing relevant concerns. More research is needed about conditions with low or medium initial treatment burden.

**International Registered Report Identifier (IRRID):**

RR2-10.2196/54833

## Introduction

### Background

Digital interventions have the potential to improve health care delivery by improving effectiveness, accessibility, the ability to reach patients, cost-effectiveness, and personalization [1-3]. The prevalent use of digital interventions and devices is constantly creating new possibilities for connecting people and providing services [4-6]. There are a variety of digital interventions used in a health care setting. In this review, we will focus on those digital interventions that facilitate and deliver health care services to patients with chronic conditions [7]. As patients directly interact with these interventions, they are the most relevant for the patient experience. The interventions can include remote appointments [8], mobile apps, wearable devices, and remote monitoring systems [9,10], electronic health records [11], and patients’ portals and online support groups [12].

Chronic conditions often require long-term management. For the patient, this can mean navigating services, interacting with health care professionals (HCPs), and adhering to treatments, which can all together create significant demands and load to the patient [13]. These demands can be referred to as treatment burden, which is defined as both the workload caused by the self-management of the condition **and** the impact the treatment has on the patient’s function and well-being [13]. In addition to the negative impact on patients’ well-being, high treatment burden is also associated with poor adherence and worse disease outcomes [14].

In this review, the terms treatment burden and burden of treatment are used interchangeably, consistent with previous literature. However, we draw specifically on the burden of treatment [14] theory, which provides a framework to explain the interactions between health care systems, patients, and their treatment burden [15]. This theoretical framework informed both our eligibility criteria and our interpretation of findings.

There are many ways digital interventions may affect treatment burden. Telehealth and self-management interventions may shift the responsibility of the care to the patient, causing additional demands [16,17], digital systems may be inaccessible or difficult to use [18], and patients may struggle with digital stress [19]. When properly designed, digital interventions can also make the health care processes simpler and more effective, reducing the treatment burden for patients. For example, remote care can reduce the time and costs needed for traveling to medical appointments [8], and self-management interventions can make self-management easier and more motivating [20,21].

### Related Work

Recent systematic reviews about the current digital interventions have shown limited efficacy and rather small benefits to patients [22,23]. Recent umbrella reviews show that most digital interventions in health care are mobile- or computer-based, the largest targeted condition group is mental illnesses, and most outcomes are focused on effectiveness [24-26]. The burden on the patient is often not considered or measured in the development and research of these interventions. On the other hand, the systematic reviews we have identified investigating the treatment burden on patients with chronic conditions [14,26-30] do not focus on digital interventions. We observe that there is a lack of systematic reviews combining these 3 concepts: treatment burden, digital interventions, and chronic conditions.

After the publication of the protocol for this review [27], 2 relevant reviews were published [28,29]. However, one of them was a scoping review, with a specific focus on telehealth [28], and the other was focused only on multimorbidity and the availability and benefits of digital tools [29]. The current systematic review will expand on these reviews by widening the scope to include all digital interventions that facilitate and deliver healthcare services to patients with a variety of chronic conditions. The summary of the related work is presented in Table 1.

**Table 1:** Summary of the related work.

Study, year	Focus of the review	Findings	Domains not addressed
Kraef et al [22], 2020	Effectiveness of digital interventions for multimorbidity.	Limited benefit of digital interventions on multimorbidity.	Treatment burden
Domhardt et al [23], 2021	Effectiveness of digital interventions for youth with chronic conditions.	Limited benefit of digital interventions on youth with chronic conditions.	Treatment burden
Taylor et al [24], 2022	Experiences of patients with chronic conditions using digital interventions.	Digital interventions are perceived positively by patients with chronic conditions.	Treatment burden is mentioned in one of the themes, but it is not the focus of the review, which is mostly focused on mental health
Xiong et al [25], 2023	Extent of use and effectiveness of digital interventions in low- and middle-income countries	Digital interventions are underused in low- and middle-income countries.	Treatment burden
Ibrahim et al [26], 2022	Mapping review studies of digital health.	Studies of digital interventions are focused on effectiveness and predominantly related to mental health interventions.	Treatment burden, mostly focused on mental health
Demain et al [14], 2015	Conceptualizing treatment burden.	Defining treatment burden and strategies for minimizing it.	Digital interventions
Alsadah et al [30], 2020	Conceptualizing treatment burden.	Defining treatment burden.	Digital interventions
Sheehan et al [31], 2019	Assessment methods of treatment burden.	Treatment burden lacks standardized assessment methods.	Digital interventions
Gallacher et al [32], 2023	Use of the burden of treatment theory.	The burden of treatment theory is an applicable tool for health care interventions.	Digital interventions
Sav et al [33], 2015	Conceptualizing treatment burden.	Conceptualizing treatment burden.	Digital interventions
Matthews et al [34], 2023	Influence of health-system change on treatment burden.	System-level interventions may affect some treatment burden domains.	Digital interventions
Tahsin et al [28], 2024	Treatment burden and telehealth for patients with chronic conditions.	Telehealth reduced travel time and costs, but caused challenges with complex health data and adapting to new technology.	A scoping review, limited to telehealth interventions
Phi et al [29], 2025	Availability and benefit of digital technologies to multimorbidity.	Less than 5% of digital interventions considered multimorbidity.	Limited to patients with multimorbidity, focusing on availability and potential benefits

### Objective

The aim of this review is to evaluate the evidence of how digital interventions impact the treatment burden experienced by people with chronic conditions, and to explore how these effects vary across different conditions and intervention types. In this review, we use the term “impact” to refer to reported or perceived effects of digital interventions on treatment burden, rather than measured causal outcomes.

### Research Questions

Based on the identified gap in research discussed above, we have defined 2 primary research questions (RQs):

- RQ1: How can digital interventions impact treatment burden on people with chronic conditions?
- RQ2: What kind of support can digital interventions provide for people with chronic conditions to lower their treatment burden?

## Methods

### Ethical Considerations

To conduct this systematic review, we followed the University of Oulu ethics process as defined in the guidelines from the Ethics Committee of Human Sciences [35]. According to the guidelines, an ethics board review was not needed.

### Design

This systematic review used a convergent design for systematic mixed studies reviews [26] to synthesize published articles describing the impact of digital interventions on patients’ treatment burden. This review is reported according to the PRISMA (Preferred Reporting Items for Systematic Reviews and Meta-Analysis) 2020 [36]. We registered the protocol on the International Prospective Register of Systematic Reviews (PROSPERO; CRD42023477605) and published the protocol describing our systematic review methodology [27].

### Information Sources and Search Strategy

The systematic search for articles was conducted, and screening was started before publishing the protocol and the detailed search strategy and inclusion criteria of this review are reported in the previously published protocol [27]. The search terms are presented in Multimedia Appendix 1. The search for articles published between January 1, 2013, and October 16, 2023, was conducted from bibliographical databases PubMed, Scopus, Web of Science, ACM, PubMed Central, and CINAHL. A supplementary search was conducted by hand searching the reference lists in systematic literature reviews and scoping reviews that were found during the literature searches. The search was done again on June 17, 2025, for papers published after October 16, 2023, to include more recent literature in the final results.

### Inclusion Criteria

We included original publications written in English and accepted in peer-reviewed journals or conference proceedings published after 2013. Although the use of digital interventions in health care for chronic conditions extends further back [37], studies before 2013 mostly refer to research prototypes not available to the public. Since then, digital health technology has developed quickly. Therefore, we decided to restrict our search to studies published after 2013 to cover all relevant publications. Qualitative, quantitative, and mixed methods articles and conference proceedings are included, whereas reviews, protocols, and book chapters were excluded.

The eligibility criteria align with the PICO (population, intervention, comparison, and outcome) framework [38]. Population: studies were eligible if participants were patients with chronic conditions, their caregivers, or HCPs involved in their care. Studies focusing solely on mental health conditions were excluded. Intervention: only studies with digital intervention for treatment or management of chronic conditions were included. Comparison: studies were neither included nor excluded based on the comparator. Outcome: only studies where the outcome was a treatment burden for the patients were included.

### Selection of Studies

We first imported the titles and abstracts of all the studies we retrieved from our search results to Covidence, a web-based screening tool [39], where duplicate records were removed. In the first stage of screening, 2 independent reviewers screened all titles and abstracts for inclusion. The Cohen κ of the consensus between reviewers (0.41 between reviewers MP and OK, and 0.59 between reviewers MP and PK) indicated moderate agreement. In the second stage, 2 reviewers screened and selected full-text papers for inclusion. The Cohen κ of the consensus in the second stage (0.32 between reviewers MP and OK, and 0.43 between reviewers MP and PK) indicated fair to moderate agreement. The Cohen κ score was calculated with the Covidence screening tool for all screened studies. Disagreements were resolved by discussion. For example, in a disagreement about whether the study included treatment burden outcomes or not, the reviewers checked the study together. The paper was included if reviewers were able to identify relevant outcomes.

A total of 36 articles were included in the data extraction stage of the initial search. From the data extraction stage, 1 study was moved back to full-text screening and removed as the intervention did not match the inclusion criteria. After data extraction, one study was removed due to a lack of participants. From the supplementary search, 5 articles were identified. One of these articles was a duplicate and therefore removed. From the second search in 2025, 8 articles were included. A total of 46 papers were included.

### Data Extraction

A data extraction form was designed using Covidence before starting the extraction. The predefined form included questions on population characteristics, study design, aims, intervention characteristics, and main results. Study designs were classified based on the tool from Grimes and Schulz [40]. The included studies covered a range of chronic conditions with varying levels of initial treatment burden, independent of the use of digital interventions. The initial treatment burden level of each condition was classified by the researchers who extracted the data (see Table 2). If explicitly stated by the original authors, the reported level of the burden was used for the classification. Otherwise, the treatment burden level was the burden to the patients. We considered a condition with high burden when the treatment is highly disruptive to the patient’s everyday life, involving more than daily medication and occasional check-ups. In cases where there was not enough information on the treatment burden level, or there were high variations between patients, we marked the treatment burden level as “Not classified.”

**Table 2:** Treatment burden level classification during data extraction.

Classification	Criteria	Example
High	The treatment is highly disruptive to the patient’s life.	Treatment routines that take hours, weekly hospital visits, and treatments that prevent working and studying.
Low or medium	The treatment of the condition is moderately or lightly disruptive to the patient’s life.	Adherence to daily medication routine, occasional check-ups.
Unclassified	Not enough information on the treatment burden level, or a high variety between patients.	The patient population includes patients with high and low treatment burden, or patients with multimorbidity, where the treatments are not known.

The data extraction was piloted beforehand; author MP extracted 5 articles, and authors PK and OK extracted 2 articles each. The authors then had a meeting to go through these extractions and suggest improvements to the data extraction table. Several adjustment needs were identified, and the data extraction form was adjusted accordingly. This included, for example, adding missing study design types and separate boxes for qualitative and quantitative results. Data extraction was performed independently by 2 reviewers. The researchers scheduled regular meetings to discuss the process. Most disagreements in consensus were resolved by reviewing the papers again. A disagreement about the treatment burden level of asthma had to be resolved during a meeting, which was decided to be low or medium after discussing together.

### Quality Assessment

The quality assessment was conducted in parallel to the data extraction*.* The Critical Appraisal tools from the Joanna Briggs Institute were used [41]. Quality assessment results are presented in the appendix (see Multimedia Appendix 2). All studies reviewed with the tool for qualitative studies had a low risk of bias. For these studies, the only topic that caused concern for bias was the lack of self-reflection, which was present in 17 out of 38 studies. Some individual studies also had unclear reporting of ethical statements, theoretical statements, and the voices of participants. For studies reviewed with an analytical cross-sectional tool, we found 2 low-risk-of-bias studies. For randomized controlled trials, all studies were high-quality, but due to the nature of the intervention, blinding of personnel and participants was missing from these studies. For the studies reviewed with the tool for quasi-experimental studies, the experiments did not have comparison groups, which causes potential bias, but other aspects of the studies were rated high quality. Overall, while the analysis revealed some sources of bias, the reviewers agreed that the included studies had credible findings.

### Data Synthesis

A convergent integrated approach was used for synthesis and integration [42]. In this approach, quantitative data is converted into qualitative data, and the qualitative and quantitative evidence are integrated and analyzed together [43]. We converted the quantitative findings into qualitative descriptions by narratively interpreting the effects reported in the quantitative studies. Examples of this conversion process are presented in Table S2 in Multimedia Appendix 3. After the conversion, both qualitative data and converted quantitative data were presented in a single dataset. With NVivo software (Lumivero), the dataset was coded line by line. An inductive coding approach was used [44]. Coded data were arranged into categories based on the digital intervention type and the reported impact on treatment burden. Examples of coding are presented in Table S3 in Multimedia Appendix 3. The coding and categorization were conducted by the first author.

## Results

**Figure 1:**
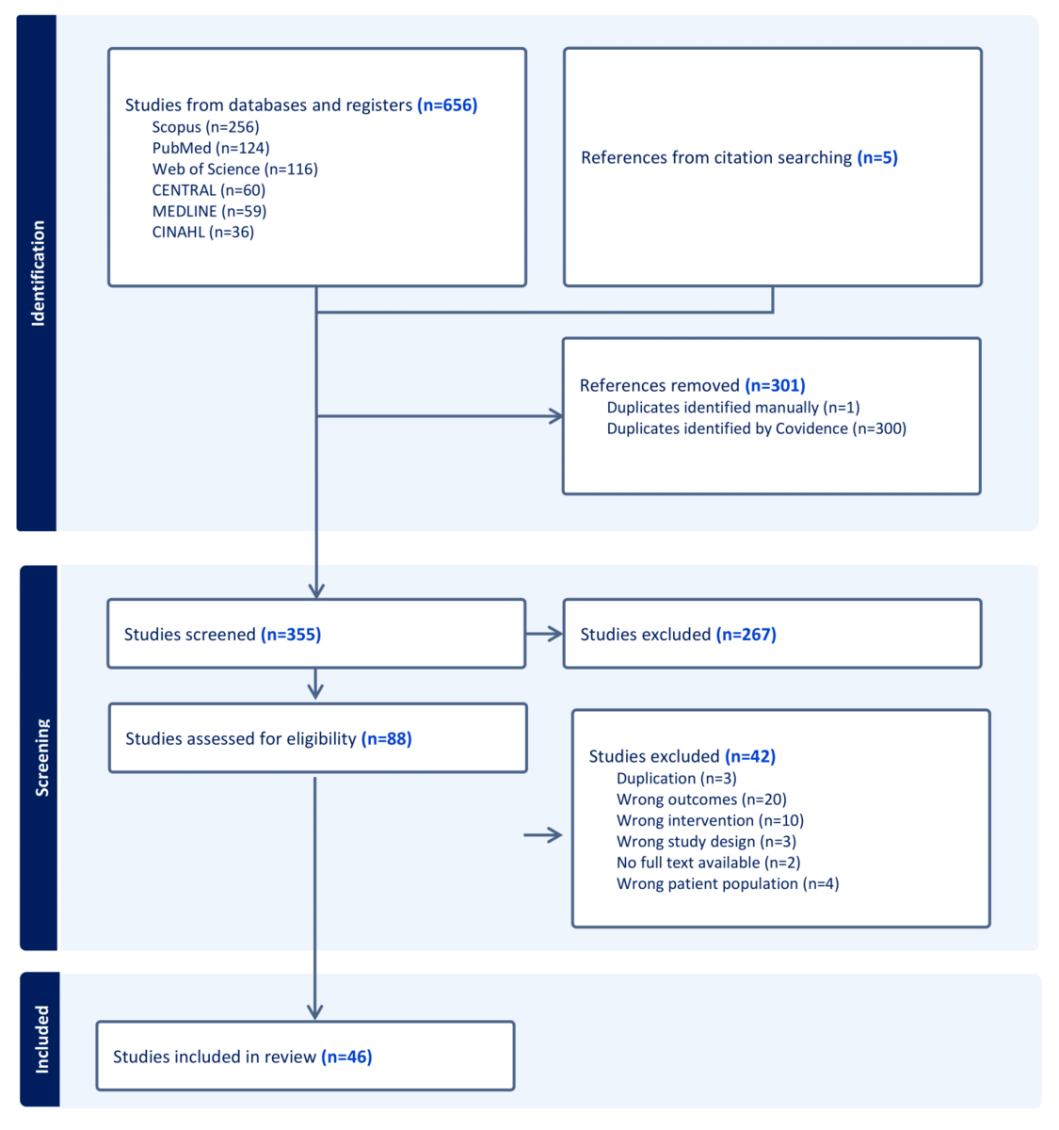
PRISMA (Preferred Reporting Items for Systematic Reviews and Meta-Analyses) chart.

In total, we found 46 studies fulfilling our inclusion criteria. The number of studies is presented in the PRISMA chart in Figure 1. Out of all the included studies, 25 were qualitative, and 12 were mixed methods. The qualitative studies interviewed patients, HCPs, caregivers, and relevant experts, with 662 interview participants in total. For all study designs, there were 4321 participants in total. Most of the studies were from Europe (n=25), and the most included conditions were chronic kidney disease (n=7), cancer (n=6), and cystic fibrosis (n=6). In 23 studies, the participants had high initial treatment burden. The details of each study are presented in Table S1 in Multimedia Appendix 3. The impacts on treatment burden are reported in Tables 3 and 4.

**Table 3:** Summary of the reported positive impacts of digital interventions on treatment burden

Identified impacts	Occurrences by article reference number	Total occurrences
Easier communication with HCPs^a^	[45-60]	16
Supporting self-management	[49-53,56,61-67]	13
Providing informational support	[45,46,54,59,68-74]	11
Increased motivation for treatment	[58,65,67,68,71,72,75,76]	8
Easy to use	[45,57,63,74,76-79]	8
High user satisfaction	[45,68,69,71,73,76,80]	7
Reducing travel	[49,50,52,59,60,66,81]	7
Saving time	[46,47,56,60,61,75,79]	7
Reduces overall treatment burden	[54,62,80,82]	4
Reduced negative feelings	[51,53,59,61,67]	5
Reduced costs	[59,74,79,83]	4
Easier communication with family	[52,69,72]	3
Empowerment	[53,63,79]	3
Reduces isolation	[46,59,70]	3
Entertainment during treatment	[66,68,73]	2
Improving accessibility	[50,61,70,75]	2
Improved quality of care	[55,56,59,65]	2
Enhances freedom	[49,63,82]	1
Acknowledgement for hard work	[62,82]	1
Reduced stigma	[59,66]	1
Reduced illness identity	[59,63]	1
Integrated health service delivery	[62]	1

^a^HCP: health care professional.

**Table 4:** Summary of the reported negative impacts of digital interventions on treatment burden.

Identified impacts	Occurrences by article reference number	Total occurrences
Time-consuming	[46,48,49,56,64,65,67,69,75,77]	10
Accessibility issues	[47,59-61,72,75,79,81,84]	9
Fear and anxiety	[45,46,56,61,67,72,81]	7
Technical problems	[45,56,60,62,79,82,84]	7
Difficult to use	[47,59,61,75,79,84]	6
Too much responsibility for the patient	[45,47,49,54,63,65]	6
Inconvenient	[47,56,60,67,69]	5
Unnecessary	[50,63,64,77]	4
Difficulties in understanding the information	[61,68,72]	3
Concerns of accuracy	[59,65,66]	3
Causing social isolation	[50,58,79]	3
Intrusive	[45,56]	2
Cost of equipment	[45,56]	2
Difficult to transport	[56,76]	2
Annoying to use	[56,76]	2
Increased illness identity	[49]	1
Could reduce the quality of care	[50]	1
Difficulties building relationships with HCPs^a^	[59]	1

^a^HCP: health care professional.

### The Impact of Different Digital Interventions on Treatment Burden (RQ1)

**Figure 2:**
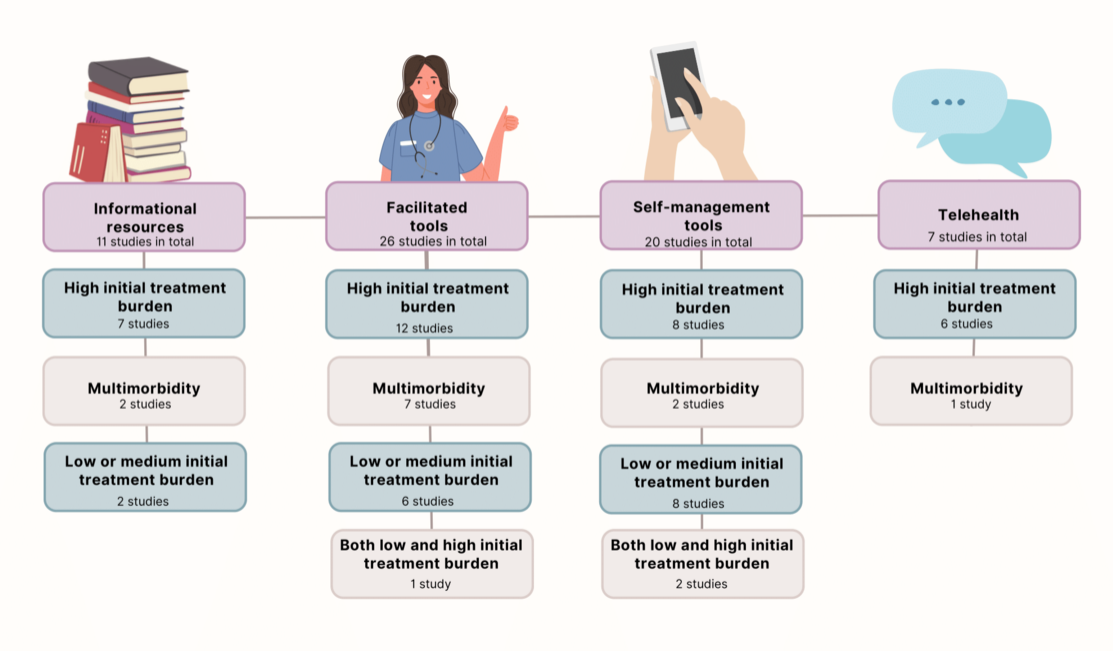
Visualization of the studies included in each intervention category.

The included studies used different types of digital interventions. We have categorized these interventions into 4 groups based on the components included in the interventions and the type of intervention. These groups are telehealth, informational resources, self-management tools, and facilitated tools. These categories are presented in Figure 2.

Telehealth refers to remote appointment portals and other tools for contacting HCPs. Informational resources cover all mobile- or web-based informational resources and decision aids provided to the patient. The self-management tools category includes all web- and mobile-based tools that the patient uses for self-management of the disease, without the help of a care team. The facilitated tools category includes the tools where an HCP or a care team is facilitating the use of the tool (for example, remote monitoring, patient portals, and digital care pathways). Some of these categories overlap. For example, digital care pathways often include many different types of components, such as remote appointment portals and informational resources.

To answer RQ1, we will present the impacts on treatment burden for each category. For the informational resources, self-management tools, and facilitated tools categories, we will discuss the impact on the conditions with high treatment burden and conditions with medium or low treatment burden. For the telehealth category, there were no studies about conditions with medium or low treatment burden, so the results will only cover conditions with high treatment burden and multimorbidity.

#### Informational Resources

The interventions in 12 studies included informational resources. A total of 2 studies had patients with low or medium initial treatment burden [65,74], 2 studies had patients with multimorbidity [85,86], and 7 had high initial treatment burden [45,46,68-72]. For all treatment burden levels, informational resources interventions were reported to have many positive impacts, the most reported being informational support. The interventions helped patients to better understand their conditions and their treatments through information delivered through websites and phone applications. Other positive impacts were easier communication with HCPs and increased motivation for treatment. The negative impacts included impacts such as difficulties in understanding the information, time-consuming tools, and fear and anxiety caused by the information. For example, information about symptoms of the conditions and invasive interventions, such as surgeries, could be frightening to people who have recently been diagnosed.

#### Facilitated Tools

In total, 27 studies mentioned tools facilitated by HCPs. A total of 7 dealt with multimorbidity [50,51,57,75,84,86,87], 6 discussed conditions with medium or low initial treatment burden [52,56,59,66,83,88], 12 high [46,48,49,60-62, 73,77,78,80,89,90], and 1 was not classified [45].

The patients with high and low or medium treatment burden levels had similar results: mostly positive impact with minor negative impact. Most reported positive impacts were supporting self-management, easier communication with HCPs, and ease of use. The functions of the tools included booking appointments and answering questionnaires online, self-monitoring markers for the condition, and other functions that the patients experienced as supportive for managing their health appointments, self-monitoring, and medication adherence. The most reported negative impacts were the difficulty of use, time-consuming tools, concerns of inaccuracy, and accessibility issues. This could be caused by, for example, inconvenient log-in procedures, time-consuming data entry tasks, and technical problems. For some patients, the traditional ways of self-managing their condition and booking appointments felt more convenient and accessible.

#### Self-Management Tools

There were 20 studies regarding self-management tools. A total of 8 studies involved conditions with medium or low initial treatment burden [53,56,58,63,65,67,76,88], 2 dealt with multimorbidity [50,75], 8 studies discussed high treatment burden [46,49,62,64,68,69,71,82], and 2 were unclassified [54,55].

For conditions with high treatment burden and multimorbidity, the most often reported benefits for treatment burden were informational support, motivation for self-management, and easier communication with HCPs. Even though the self-management tools are not used to directly contact HCPs, they can help patients to understand their own condition better, and track and measure their symptoms, which can also make it easier to communicate issues to HCPs. The most reported negative impacts on treatment burden were time-consuming tools and adding responsibility to patients. When using self-management tools, there is no option of contacting members from the health care team directly, and the use is not facilitated or observed by HCPs. For many patients, this may cause additional burden, and they may have difficulties using these tools by themselves.

The self-management tools for low or medium initial treatment burden were the only category where impacts on treatment burden were reported to be more negative than positive. The most commonly reported negative impacts were adding responsibility to patients and being unnecessary, and too time-consuming. The studies mentioned that the positive impacts were self-management tools were easy to use, motivation for self-management, and reassurance.

#### Telehealth

There were 8 studies regarding telehealth. One study was about multimorbidity [50], and 6 were about conditions with high treatment burden [46,47,61,62,79,81]. Telehealth had mostly positive impacts, such as supporting self-management, saving time, and improving communication with HCPs. Online appointments can reduce the time spent traveling to the hospital, and messaging systems can reduce the time spent queuing on the phone. For negative impacts, inaccessibility, technical challenges, and difficulty of use were the most often reported. Inconvenient login procedures, bad network connections, and difficulties in using the messaging and video call platforms were some of the problems mentioned. A study mentioned that negative impacts were only present when starting to use the intervention and disappeared once familiar with the telehealth system.

### Supporting Patients With Treatment Burden (RQ2)

To answer RQ2, we categorized all the identified impacts into 4 main ways digital interventions can support patients with their treatment burden. These include providing support for improving access to health care, self-management, improving user experience, and addressing relevant concerns. Next, we will go through these categories in more detail.

#### Improving Access to Health Care

The most discussed theme was access to health care and communication to HCPs [45-60]. Digital interventions have the potential to make health care more accessible and offer easier and better ways to contact HCPs. However, digital interventions can themselves also be inaccessible [47,59-61,72,75,79,81,84], for example, to individuals with low digital literacy, reduced hand mobility, or those who do not own digital devices. Inaccessibility is particularly problematic for those interventions that replace nondigital alternatives, such as telehealth options instead of using mail and face-to-face appointments for communication. This can explain why the telehealth and facilitated tools categories in our review were divided in opinions. Some studies report that they are easy to use and increase access to care, but there are also concerns that they are difficult to use and inaccessible. This suggests that there are two groups of people: those who would benefit from telehealth interventions, and those for whom they are not suitable. Therefore, it is important to keep non-digital options for accessing health care available while developing digital interventions for those who benefit from them.

#### Providing Support for Self-Management

The second most brought up theme was support for self-management [49-53,56,61-67]. Digital interventions have the potential to support patients with their self-management by providing them with relevant information [45,46,54,59,68-74], increasing motivation for self-management [58,65,67,68,71,72,75,76], for example, by making self-management easier and more entertaining. However, the downside of interventions focusing on self-management is that they may add the responsibility of care to patients [45,47,49,54,63,65] and cause negative emotions such as fear and anxiety [45,46,56,61,67,72,81]. For patients with low or medium treatment burden, self-management tools had a more negative impact on the treatment burden. Patients with lower treatment burden have less need for self-management, so the benefit they gain from these interventions may not be worth the added responsibility and other negative impacts. Self-management interventions should therefore be targeted to user groups that have real self-management needs, and for whom the benefits will outweigh the harms.

#### Improving User Experience

A few studies reported that digital interventions save time [46,47,56,60,61,75,79], reduce travel [49,50,52,59,60,66,81], and are easy to use [45,57,63,76-79], improving the patient’s experience. Many studies, however, reported that there was an additional burden caused by issues with the user experience of the existing digital interventions. The interventions were reported to be difficult to use [47,59,61,75,79,84], time-consuming [46,48,49,56,64,65,67,69,75,77], annoying [56,76], intrusive [45,56], difficult to transport [56,76], and cause technical problems [45,56,60,62,79,82,84]. This burden could be minimized by improving the user experience and usability of the digital interventions.

#### Addressing Relevant Concerns

Digital interventions have the potential to effectively address patients’ concerns that are difficult to traditional care, since digital interventions can be used at any time from one’s home. Several studies reported benefits of digital interventions for supporting communication with family [52,69,72], reducing social isolation [46,59,70] and negative feelings such as fear and anxiety [51,53,59,61,67], providing empowerment [53,63,79], and acknowledgment for hard work [62]. Digital interventions that can replace face-to-face care could also reduce patients’ illness identity [59] and the costs of health care [59,74,79,83] as well as enhance freedom [82]. It is important to consider, however, that all these concerns do not apply to all patients, and digital interventions should be designed to acknowledge those concerns that are relevant to the patient group using that intervention.

## Discussion

### Principal Findings

This review investigated the impact of digital health care on the treatment burden of patients with chronic conditions. Digital interventions appear to impact on the treatment burden of people with chronic illness, for those whose illness generate high to medium burden the impact was positive on all technology categories, and some negative impact related mostly to time-consuming tools, accessibility issues, and difficulties of using tools The impact in people with low and medium treatment burden related to their chronic illness was mostly positive in other technology categories, but self-management tools had negative impact on the treatment burden, as they were often considered unnecessary and added responsibility to the patients.

We found that informational support had very little negative impact and many positive impacts. This may imply that it is mostly a suitable way to provide information to patients, and there are no major concerns in terms of treatment burden. Telehealth and facilitated tools seem to divide opinions, and self-management tools without support from HCPs had a relatively more negative impact.

Overall, digital interventions can help patients with their treatment burden by supporting self-management, improving access to health care, improving patients’ experience, and addressing relevant concerns.

### Comparison With Previous Work

Our findings were consistent with previous literature [8,20,21] in terms of finding the main positive impacts on supporting self-management, supporting communication with HCPs, and saving time and travel. We also found issues with accessibility and difficulty of using the tools and increased responsibility to patients, as in the previous literature on the negative impact of digital interventions [16-18].

The Burden of Treatment theory framework [15] about the relationships between patients’ treatment burden and the health care system in general is also consistent with our findings about digital interventions. The quality of digital interventions and the support available from HCPs directly impact the treatment burden experienced by the patient. Digital interventions have the potential to both reduce and increase the patient’s health care workload, which in turn affects the patient’s capacity to take responsibility for their care.

### Strengths and Limitations

This is a novel review that covers the impact of digital health care on the treatment burden of patients. Our novel findings were related to the differences in impact on different types of digital interventions. We found that there are negative impacts for the group of patients who have conditions with low or medium treatment burden using self-management tools. This may be caused by people with higher treatment burdens having more needs for support and therefore gaining more benefit from interventions supporting the treatment. Self-management tools are also more problematic than facilitated tools in terms of patients’ treatment burden, as the sole responsibility is on the patient, and professional help is not available.

We have found novel findings demonstrating how treatment burden is a relevant concept for future digital health care research. Our findings show how the initial treatment burden of the patient’s condition and the type of digital intervention both have an impact on the benefits and harms of using the intervention and, therefore, may impact the overall success and usage of the intervention.

This review also has some limitations. First, our search was restricted to studies published in English. This may have excluded relevant papers from developing regions where English is not the primary language for scientific publication*.* Although we attempted to conduct thorough searches from 6 different databases, the included studies were mostly from Europe and North America, and from countries with high economic and digitalization levels*.* A recent scoping review found that digital interventions are harder to integrate in low-resource communities, and the experience of treatment burden can be different for marginalized groups [28]. Patients of low socioeconomic status are less likely to be offered digital interventions [91], and often live in areas that have poor internet infrastructure, causing more barriers and difficulties accessing digital interventions [92]. There is a need for future research focusing on the equity and treatment burden experience of structurally marginalized groups and patients living in low-resource areas. Programs should be developed to respond to the needs of these patient groups and address the accessibility issues.

Second, most of the included studies were investigating conditions with high burden or multimorbidity. We included a variety of conditions in the search terms, but especially the papers about telehealth and informational resources were missing research on conditions with low or medium treatment burden. Therefore, our findings about telehealth and informational resources may not apply to this patient population.

### Future Directions

We found that some intervention types have more concerns than others, and some are not suitable for all patient groups. This can be useful information for the designers of digital interventions to design better interventions. HCPs can also target suitable interventions for the right patient populations. We have also identified a research gap concerning conditions with low or medium treatment burden. Treatment burden is also a relevant concept for these groups of patients, and future research should address this gap.

### Conclusions

The results of this study indicate that digital interventions mostly support patients with chronic conditions with their treatment burden. The main benefits are support with self-management, informational support, and easier ways to contact HCPs. The main concerns were accessibility issues, time-consuming tools, and causing fear and anxiety. Future research about conditions with low or medium initial treatment burden would be needed to better understand the impact of digital solutions on patients’ treatment burden.

## Appendix

Multimedia Appendix 1 Full search terms.

Multimedia Appendix 2 Quality assessment tables.

Multimedia Appendix 3 Summary of studies.

Multimedia Appendix 4 PRISMA checklist.

## Abbreviations

HCP: health care professional
PICO: population, intervention, comparison, and outcome
PRISMA: Preferred Reporting Items for Systematic Reviews and Meta-Analyses
PROSPERO: International Prospective Register of Systematic Reviews
RQ: research question

## Data Availability

Data sharing is not applicable to this article as no datasets were generated or analyzed during this study.
