# Supplementary material for: Impact of Digital Interventions on the Treatment Burden of Patients With Chronic Conditions: Systematic Review
Source: J Med Internet Res. 2025 Nov 21;27:e66874. doi: 10.2196/66874 (PMC12680937; doi:10.2196/66874)
Supplement: Multimedia Appendix 2 [file jmir_v27i1e66874_app2.docx]

Table S1. Quality assessment for the qualitative studies.

|  | **Philosophical perspective** | **Objectives** | **Data collection** | **Data analysis** | **Interpretation of results** | **Theoretical statement** | **Self-reflection** | **Participants' voices** | **Ethical approval** | **Conclusions** |
| --- | --- | --- | --- | --- | --- | --- | --- | --- | --- | --- |
| Fairbrother et al., 2014 [45] |  | 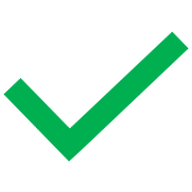 | 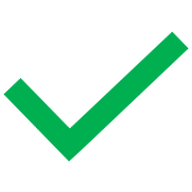 | 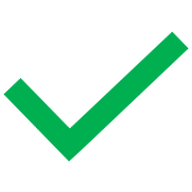 | 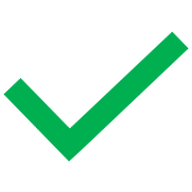 | 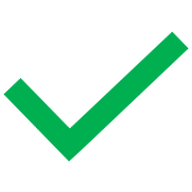 | 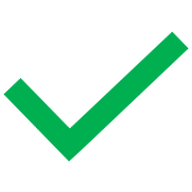 | 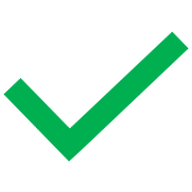 | 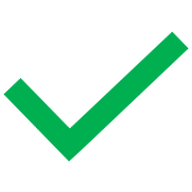 | 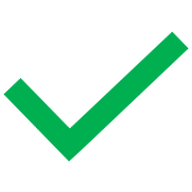 |
| Högberg et al., 2013 [47] |  | 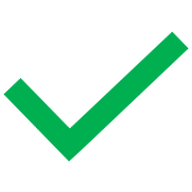 | 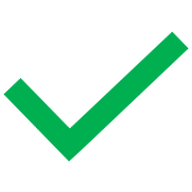 | 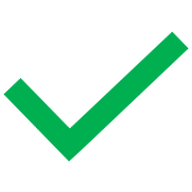 | 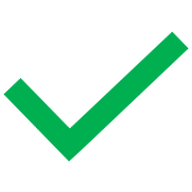 | 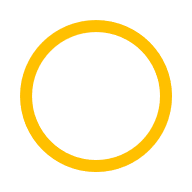 | 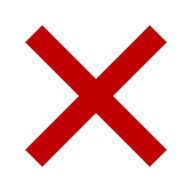 | 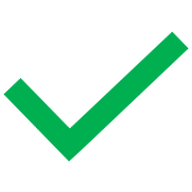 | 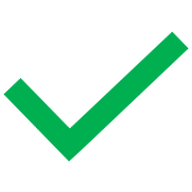 | 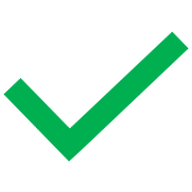 |
| Hilliard et al., 2014 [46] |  | 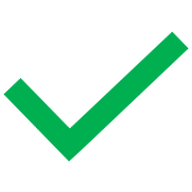 | 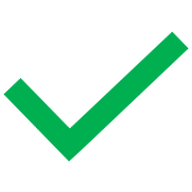 | 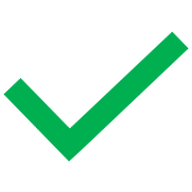 | 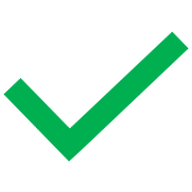 |  |  | 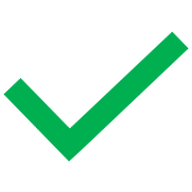 | 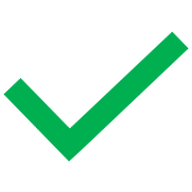 | 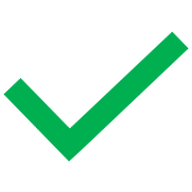 |
| Fergus et al., 2014 [69] |  | 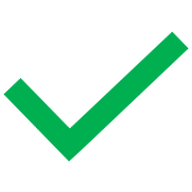 | 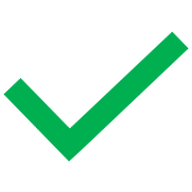 | 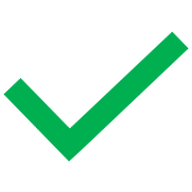 | 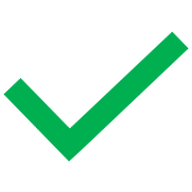 | 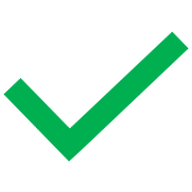 | 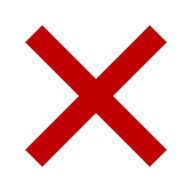 | 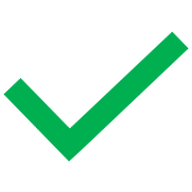 | 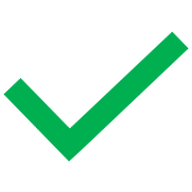 | 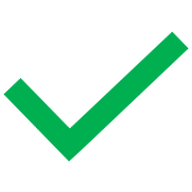 |
| Sabesan et al., 2014 [89] |  | 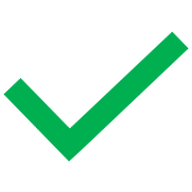 | 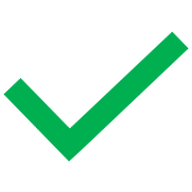 | 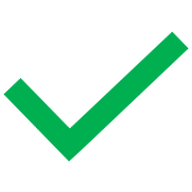 | 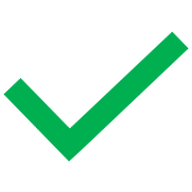 | 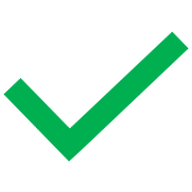 | 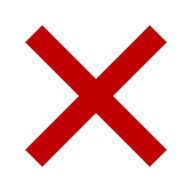 | 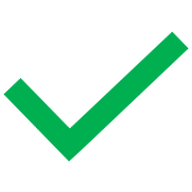 | 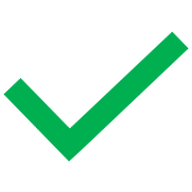 | 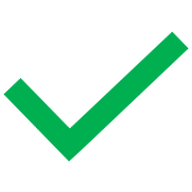 |
| Doyle et al., 2019 [86] |  | 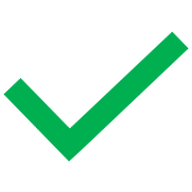 | 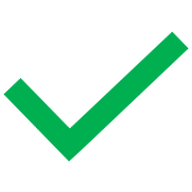 | 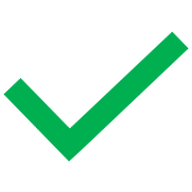 | 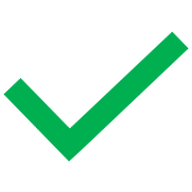 | 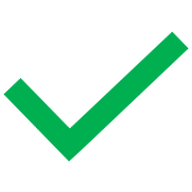 | 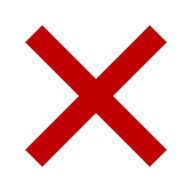 | 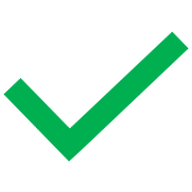 | 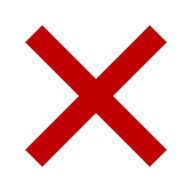 | 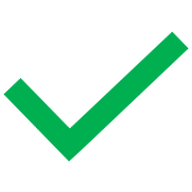 |
| Ryu et al., 2023 [51] |  | 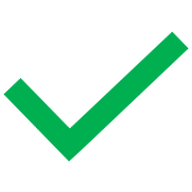 | 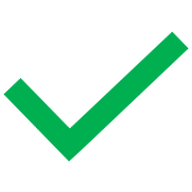 | 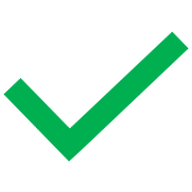 | 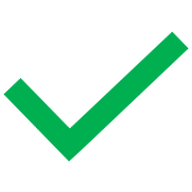 | 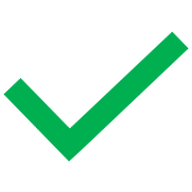 | 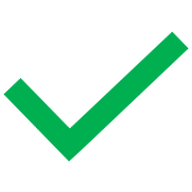 | 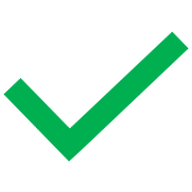 | 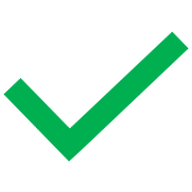 | 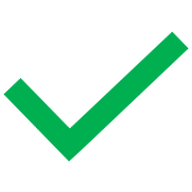 |
| Doyle et al., 2017 [85] |  | 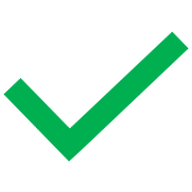 | 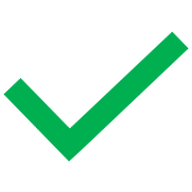 | 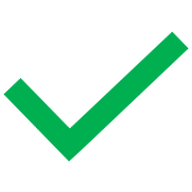 | 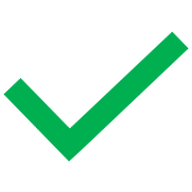 | 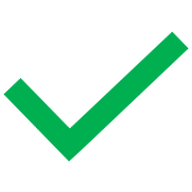 | 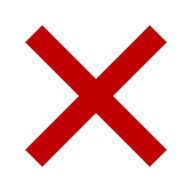 | 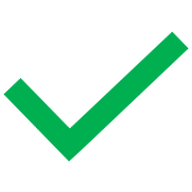 | 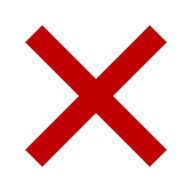 | 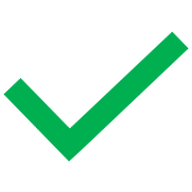 |
| Ongwere et al., 2018 [87] |  | 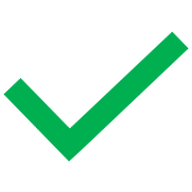 | 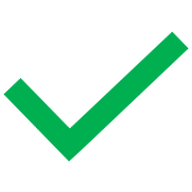 | 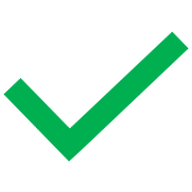 | 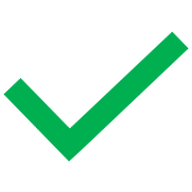 | 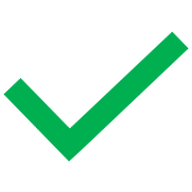 | 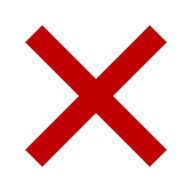 | 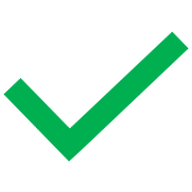 | 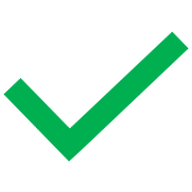 | 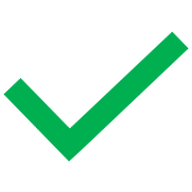 |
| Pichon et al., 2021 [53] |  | 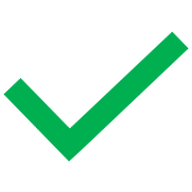 | 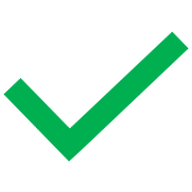 | 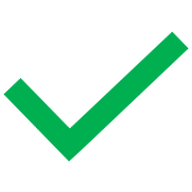 | 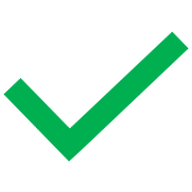 | 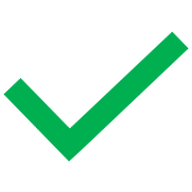 | 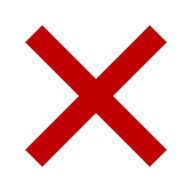 | 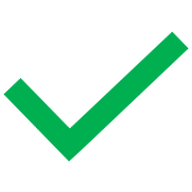 | 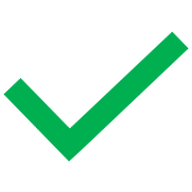 | 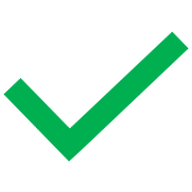 |
| Isika et al., 2020 [74] |  | 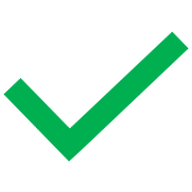 | 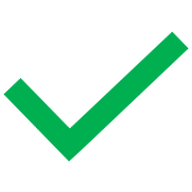 | 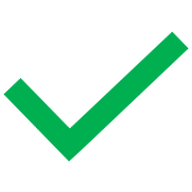 | 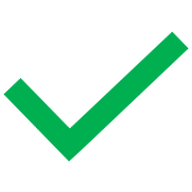 | 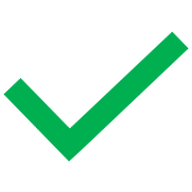 | 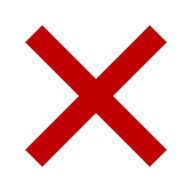 | 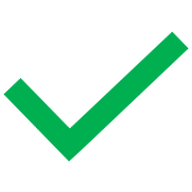 | 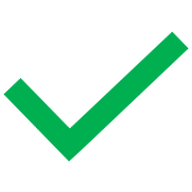 | 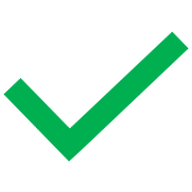 |
| Rodger et al., 2019 [49] |  | 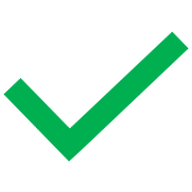 | 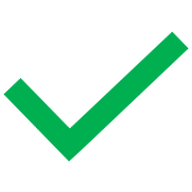 | 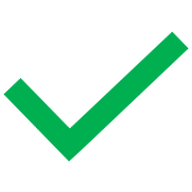 |  |  |  |  |  |  |
| Raaijmakers et al., 2022 [75] |  |  |  |  |  |  |  |  |  |  |
| Knowles et al., 2021 [48] |  |  |  |  |  |  |  |  |  |  |
| Cleres et al., 2021 [76] |  |  |  |  |  |  |  |  |  |  |
| Bouamrane et al., 2019 [73] |  |  |  |  |  |  |  |  |  |  |
| Morton et al., 2018 [67] |  |  |  |  |  |  |  |  |  |  |
| Meiklem et al., 2021 [89] |  |  |  |  |  |  |  |  |  |  |
| Meiklem et al., 2022 [78] |  |  |  |  |  |  |  |  |  |  |
| Stringer et al., 2023 [72] |  |  |  |  |  |  |  |  |  |  |
| Schrader et al., 2014 [84] |  |  |  |  |  |  |  |  |  |  |
| Skovlund et al., 2023 [61] |  |  |  |  |  |  |  |  |  |  |
| van Schelven et al., 2023 [55] |  |  |  |  |  |  |  |  |  |  |
| Walsh et al., 2019 [52] |  |  |  |  |  |  |  |  |  |  |
| Runz-Jørgensen et al., 2017 [50] |  |  |  |  |  |  |  |  |  |  |
| Huuskes et al., 2021 [79] |  |  |  |  |  |  |  |  |  |  |
| McBride et al., 2020 [63] |  |  |  |  |  |  |  |  |  |  |
| Tran et al., 2019 [54] |  |  |  |  |  |  |  |  |  |  |
| Flythe et al., 2022 [82] |  |  |  |  |  |  |  |  |  |  |
| Spencer-Bonilla et al., 2021 [88] |  |  |  |  |  |  |  |  |  |  |
| Al Zahidy et al., 2025 [56] |  |  |  |  |  |  |  |  |  |  |
| Betsema et al., 2025 [64] |  |  |  |  |  |  |  |  |  |  |
| Duckworth et al., 2024 [65] |  |  |  |  |  |  |  |  |  |  |
| Eton et al., 2024 [13] |  |  |  |  |  |  |  |  |  |  |
| Luc et al., 2025 [58] |  |  |  |  |  |  |  |  |  |  |
| Polus et al., 2024 [59] |  |  |  |  |  |  |  |  |  |  |
| Shewamene et al., 2024 [60] |  |  |  |  |  |  |  |  |  |  |
| Wali et al., 2023 [66] |  |  |  |  |  |  |  |  |  |  |

Table S2. Quality assessment for the randomized controlled trials.

|  | **Group assignment** | **Concealed group allocation** | **Similar baseline** | **Blinded participants** | **Blinded researchers** | **Identical treatment of groups** | **Blinded outcome assessors** | **Outcome measurement** | **Reliability of measurements** | **Follow-up** | **Analysis in groups** | **Appropriate statistical analysis** | **Trial design** |
| --- | --- | --- | --- | --- | --- | --- | --- | --- | --- | --- | --- | --- | --- |
| Wildman et al., 2022 [90] |  |  |  |  |  |  |  |  |  |  |  |  |  |
| Hale et al., 2023 [83] |  |  |  |  |  |  |  |  |  |  |  |  |  |
| Uchiyama et al., 2022 [80] |  |  |  |  |  |  |  |  |  |  |  |  |  |

|  | Inclusion criteria | Subjects and settings | Intervention measures | Condition measures | Idnetifying bias | Mitigating bias | Outcome measures | Data analysis |
| --- | --- | --- | --- | --- | --- | --- | --- | --- |
| Jung et al., 2021 [70] |  |  |  |  |  |  |  |  |
| Dhingra et al., 2020 [77] |  |  |  |  |  |  |  |  |

Table S3. Quality assessment for the analytic cross-sectional studies.

Table S4. Quality assessment for the non-randomized experimental studies.

|  | **Clarity of cause and effect** | **Comparisons of participants** | **Comparisons with similar treatment/care** | **Control group** | **Multiple measurements of outcome** | **Follow-up** | **Comparisons of outcomes** | **Outcome measurements** | **Appropriate statistical analysis** |
| --- | --- | --- | --- | --- | --- | --- | --- | --- | --- |
| Robinson et al., 2020 [71] |  |  |  |  |  |  |  |  |  |
| Chai et al., 2022 [68] |  |  |  |  |  |  |  |  |  |
| Watanabe et al., 2023 [62] |  |  |  |  |  |  |  |  |  |

References:

[13] D. Eton *et al.*, “Building a measurement framework of burden of treatment in complex patients with chronic conditions: a qualitative study,” *Patient Relat Outcome Meas*, p. 39, Aug. 2012, doi: 10.2147/PROM.S34681.

[45] P. Fairbrother *et al.*, “Telemonitoring for chronic heart failure: the views of patients and healthcare professionals – a qualitative study,” *J Clin Nurs*, vol. 23, no. 1–2, pp. 132–144, 2014, doi: <https://doi.org/10.1111/jocn.12137>.

[46] M. E. Hilliard, A. Hahn, A. K. Ridge, M. N. Eakin, and K. A. Riekert, “User Preferences and Design Recommendations for an mHealth App to Promote Cystic Fibrosis Self-Management,” *JMIR mHealth uHealth*, vol. 2, no. 4, pp. e44-, 2014, doi: 10.2196/mhealth.3599.

[47] K. Högberg, L. Sandman, M. Nyström, D. Stockelberg, and A. Broström, “Prerequisites required for the provision and use of web-based communication for psychosocial support in haematologic care,” *European Journal of Oncology Nursing*, vol. 17, no. 5, pp. 596–602, 2013, doi: <https://doi.org/10.1016/j.ejon.2013.01.005>.

[48] S. E. Knowles, A. Ercia, F. Caskey, M. Rees, K. Farrington, and S. N. Van der Veer, “Participatory co-design and normalisation process theory with staff and patients to implement digital ways of working into routine care: the example of electronic patient-reported outcomes in UK renal services.,” *BMC Health Serv Res*, vol. 21, no. 1, pp. 1–11, 2021, doi: 10.1186/s12913-021-06702-y.

[49] S. Rodger and K. O’Hara, “Exploring the Potential for Technology to Improve Cystic Fibrosis Care Provision: Patient and Professional Perspectives,” *Proc. ACM Hum.-Comput. Interact.*, vol. 3, no. CSCW, p., 2019, doi: 10.1145/3359223.

[50] S. M. Runz-Jørgensen, M. L. Schiøtz, and U. Christensen, “Perceived value of eHealth among people living with multimorbidity: a qualitative study.,” *J Comorb*, vol. 7, no. 1, pp. 96–111, 2017, doi: 10.15256/joc.2017.7.98.

[51] H. Ryu *et al.*, “‘You Can See the Connections’: Facilitating Visualization of Care Priorities in People Living with Multiple Chronic Health Conditions,” in *Proceedings of the 2023 CHI Conference on Human Factors in Computing Systems*, New York, NY, USA: Association for Computing Machinery, 2023, p. doi: 10.1145/3544548.3580908.

[52] A. Walsh *et al.*, “Real-time data monitoring for ulcerative colitis: patient perception and qualitative analysis.,” *Intest Res*, vol. 17, no. 3, pp. 365–374, 2019, doi: 10.5217/ir.2018.00173.

[53] A. Pichon *et al.*, “Divided We Stand: The Collaborative Work of Patients and Providers in an Enigmatic Chronic Disease,” *Proc. ACM Hum.-Comput. Interact.*, vol. 4, no. CSCW3, p., 2021, doi: 10.1145/3434170.

[54] V. T. Tran, C. Riveros, and P. Ravaud, “Patients’ views of wearable devices and AI in healthcare: findings from the ComPaRe e-cohort.,” *NPJ Digit Med*, vol. 2, p. 53, 2019, doi: 10.1038/s41746-019-0132-y.

[55] van S. F, van der M. E, E. Wessels, and H. R. Boeije, “Let Us Talk Treatment: Using a Digital Body Map Tool to Examine Treatment Burden and Coping Strategies Among Young People with a Chronic Condition.,” *Patient Prefer Adherence*, vol. 17, pp. 517–529, 2023, doi: 10.2147/PPA.S400702.

[56] M. A. Al Zahidy *et al.*, “Digital Medicine Tools and the Work of Being a Patient: A Qualitative Investigation of Digital Treatment Burden in Patients With Diabetes,” *Mayo Clinic Proceedings: Digital Health*, vol. 3, no. 1, p. 100180, Mar. 2025, doi: 10.1016/j.mcpdig.2024.11.001.

[58] A. Luc, N. Lambricht, I. Aujoulat, C. Detrembleur, and L. Pitance, “Experiences of People With Persistent Nonspecific Neck Pain Who Used Immersive Virtual Reality Serious Games in the Home Setting: A Qualitative Study,” *Phys Ther*, vol. 105, no. 3, Mar. 2025, doi: 10.1093/ptj/pzae149.

[59] M. Polus *et al.*, “The Role of Digital Care Pathway for Epilepsy on Patients’ Treatment Burden: Clinicians’ Perspective,” 2024, pp. 257–268. doi: 10.1007/978-3-031-59080-1_19.

[60] Z. Shewamene *et al.*, “Facilitators and barriers to uptake of digital adherence technologies in improving TB care in Ethiopia: A qualitative study,” *PLOS Digital Health*, vol. 3, no. 11, p. e0000667, Nov. 2024, doi: 10.1371/journal.pdig.0000667.

[61] S. E. Skovlund, S. Renza, J. Laurent, and P. Cerletti, “Identification of Core Outcome Domains and Design of a Survey Questionnaire to Evaluate Impacts of Digital Health Solutions That Matter to People With Diabetes.,” *J Diabetes Sci Technol*, pp. 19322968231179740-, 2023, doi: 10.1177/19322968231179740.

[62] A. H. Watanabe *et al.*, “Patient Perspectives on the Use of Digital Technology to Help Manage Cystic Fibrosis.,” *Pulm Med*, vol. 2023, p. 5082499, 2023, doi: 10.1155/2023/5082499.

[63] C. M. McBride, E. C. Morrissey, and G. J. Molloy, “Patients’ Experiences of Using Smartphone Apps to Support Self-Management and Improve Medication Adherence in Hypertension: Qualitative Study.,” *JMIR Mhealth Uhealth*, vol. 8, no. 10, pp. e17470-, 2020, doi: 10.2196/17470.

[64] L. Betsema, M. Yang, A. Bohr, A. Herrera, and S. Kaae, “Cystic fibrosis patients’ preferences for electronic devices that monitor their inhalation – A qualitative study,” *Respir Med*, vol. 238, p. 107980, Mar. 2025, doi: 10.1016/j.rmed.2025.107980.

[65] C. Duckworth *et al.*, “Characterising user engagement with mHealth for chronic disease self-management and impact on machine learning performance,” *NPJ Digit Med*, vol. 7, no. 1, p. 66, Mar. 2024, doi: 10.1038/s41746-024-01063-2.

[66] S. Wali, A. Remtulla Tharani, D. Balmer-Minnes, J. A. Cafazzo, J. Laks, and A. Jeewa, “Exploring the use of a digital therapeutic intervention to support the pediatric cardiac care journey: Qualitative study on clinician perspectives,” *PLOS Digital Health*, vol. 2, no. 12, p. e0000371, Dec. 2023, doi: 10.1371/journal.pdig.0000371.

[67] K. Morton *et al.*, “Qualitative process study to explore the perceived burdens and benefits of a digital intervention for self-managing high blood pressure in Primary Care in the UK,” *BMJ Open*, vol. 8, no. 5, p., 2018, doi: 10.1136/bmjopen-2017-020843.

[68] C. W. E. Chai, B. T. Lau, M. K. T. Tee, and A. Al Mahmud, “Evaluating a serious game to improve childhood cancer patients’ treatment adherence,” *Digit Health*, vol. 8, p., 2022, doi: 10.1177/20552076221134457.

[69] K. D. Fergus *et al.*, “Development and pilot testing of an online intervention to support young couples’ coping and adjustment to breast cancer,” *Eur J Cancer Care (Engl)*, vol. 23, no. 4, pp. 481–492, 2014, doi: <https://doi.org/10.1111/ecc.12162>.

[70] A. Jung *et al.*, “Well-being and Perceptions of Supportive Resources among Caregivers of Patients with Bladder Cancer,” *Bladder Cancer*, vol. 7, no. 1, pp. 43–52, 2021, doi: 10.3233/BLC-200412.

[71] N. L. Robinson, J. Connolly, L. Hides, and D. J. Kavanagh, “A Social Robot to Deliver an 8-Week Intervention for Diabetes Management: Initial Test of Feasibility in a Hospital Clinic,” in *Social Robotics: 12th International Conference, ICSR 2020, Golden, CO, USA, November 14–18, 2020, Proceedings*, Berlin, Heidelberg: Springer-Verlag, 2020, pp. 628–639. doi: 10.1007/978-3-030-62056-1_52.

[72] E. Stringer, J. J. Lum, J. Livergant, and A. W. Kushniruk, “Decision Aids for Patients With Head and Neck Cancer: Qualitative Elicitation of Design Recommendations From Patient End Users,” *JMIR Hum Factors*, vol. 10, p., 2023, doi: 10.2196/43551.

[73] M.-M. Bouamrane *et al.*, “Haemodialysis electronic patient portal: A design requirements analysis and feasibility study with domain experts,” in *Proceedings - IEEE Symposium on Computer-Based Medical Systems*, 2019, pp. 212–216. doi: 10.1109/CBMS.2019.00051.

[74] N. Isika, A. Mendoza, and R. Bosua, “‘I Need to Compartmentalize Myself’: Appropriation of Instagram for Chronic Illness Management,” in *Proceedings of the Australasian Computer Science Week Multiconference*, New York, NY, USA: Association for Computing Machinery, 2020, p. doi: 10.1145/3373017.3373040.

[75] L. Raaijmakers, J. Vercoulen, T. Schermer, and E. Bischoff, “OPTIMA FORMA - Towards a patient-centred multimorbidity approach for chronic disease management in primary care...22nd International Conference on Integrated Care, May 23-25, 2022, Odense, Denmark.,” *International Journal of Integrated Care (IJIC)*, vol. 22, pp. 1–2, doi: 10.5334/ijic.ICIC22014.

[76] D. Cleres, F. Rassouli, M. Brutsche, T. Kowatsch, and F. Barata, “Lena: A Voice-Based Conversational Agent for Remote Patient Monitoring in Chronic Obstructive Pulmonary Disease,” in *CEUR Workshop Proceedings*, 2021, p. [Online]. Available: https://www.scopus.com/inward/record.uri?eid=2-s2.0-85110528552&partnerID=40&md5=c89b4cda5b6594a6adf6da4ba1eba898

[77] L. Dhingra *et al.*, “Addressing the burden of illness in adults with cystic fibrosis with screening and triage: An early intervention model of palliative care.,” *J Cyst Fibros*, vol. 19, no. 2, pp. 262–270, 2020, doi: 10.1016/j.jcf.2019.08.009.

[78] R. Meiklem *et al.*, “Patients’ and Clinicians’ Perspectives on the Acceptability of Completing Digital Quality of Life Questionnaires During Routine Haemodialysis Clinics: A Mixed-Methods Study,” in *Studies in Health Technology and Informatics*, 2022, pp. 752–756. doi: 10.3233/SHTI220179.

[79] B. M. Huuskes *et al.*, “Kidney transplant recipient perspectives on telehealth during the COVID-19 pandemic.,” *Transpl Int*, vol. 34, no. 8, pp. 1517–1529, 2021, doi: 10.1111/tri.13934.

[80] K. Uchiyama *et al.*, “Effects of a remote patient monitoring system for patients on automated peritoneal dialysis: a randomized crossover controlled trial.,” *Int Urol Nephrol*, vol. 54, no. 10, pp. 2673–2681, 2022, doi: 10.1007/s11255-022-03178-5.

[81] S. Sabesan, J. Kelly, R. Evans, and S. Larkins, “A tele-oncology model replacing face-to-face specialist cancer care: perspectives of patients in North Queensland,” *J Telemed Telecare*, vol. 20, no. 4, pp. 207–211, 2014, doi: 10.1177/1357633X14529237.

[82] J. E. Flythe *et al.*, “Development of a Patient Preference Survey for Wearable Kidney Replacement Therapy Devices.,” *Kidney360*, vol. 3, no. 7, pp. 1197–1209, 2022, doi: 10.34067/KID.0001862022.

[83] E. M. Hale *et al.*, “Use of digital measurement of medication adherence and lung function to guide the management of uncontrolled asthma (INCA Sun): a multicentre, single-blinded, randomised clinical trial.,” *Lancet Respir Med*, vol. 11, no. 7, pp. 591–601, 2023, doi: 10.1016/S2213-2600(22)00534-3.

[84] G. Schrader *et al.*, “An eHealth Intervention for Patients in Rural Areas: Preliminary Findings From a Pilot Feasibility Study.,” *JMIR Res Protoc*, vol. 3, no. 2, pp. e27-, 2014, doi: 10.2196/resprot.2861.

[85] J. Doyle *et al.*, “Addressing Medication Management for Older People with Multimorbidities: A Multi-Stakeholder Approach,” in *Proceedings of the 11th EAI International Conference on Pervasive Computing Technologies for Healthcare*, New York, NY, USA: Association for Computing Machinery, 2017, pp. 78–87. doi: 10.1145/3154862.3154883.

[86] J. Doyle *et al.*, “Managing Multimorbidity: Identifying Design Requirements for a Digital Self-Management Tool to Support Older Adults with Multiple Chronic Conditions,” in *Proceedings of the 2019 CHI Conference on Human Factors in Computing Systems*, New York, NY, USA: Association for Computing Machinery, 2019, pp. 1–14. doi: 10.1145/3290605.3300629.

[87] T. Ongwere, G. Cantor, S. R. Martin, P. C. Shih, J. Clawson, and K. Connelly, “Design Hotspots for Care of Discordant Chronic Comorbidities: Patients’ Perspectives,” in *Proceedings of the 10th Nordic Conference on Human-Computer Interaction*, New York, NY, USA: Association for Computing Machinery, 2018, pp. 571–583. doi: 10.1145/3240167.3240221.

[88] G. Spencer-Bonilla *et al.*, “Patient Work and Treatment Burden in Type 2 Diabetes: A Mixed-Methods Study.,” *Mayo Clin Proc Innov Qual Outcomes*, vol. 5, no. 2, pp. 359–367, 2021, doi: 10.1016/j.mayocpiqo.2021.01.006.

[89] R. Meiklem *et al.*, “Advanced Kidney Disease Patient Portal: Implementation and Evaluation with Haemodialysis Patients,” in *Lecture Notes in Computer Science (including subseries Lecture Notes in Artificial Intelligence and Lecture Notes in Bioinformatics)*, 2021, pp. 175–196. doi: 10.1007/978-3-030-85616-8_12.

[90] M. J. Wildman *et al.*, “Self-management intervention to reduce pulmonary exacerbations by supporting treatment adherence in adults with cystic fibrosis: a randomised controlled trial.,” *Thorax*, vol. 77, no. 5, pp. 461–469, 2022, doi: 10.1136/thoraxjnl-2021-217594.
