# Supplementary material for: Impact of Digital Interventions on the Treatment Burden of Patients With Chronic Conditions: Systematic Review
Source: J Med Internet Res. 2025 Nov 21;27:e66874. doi: 10.2196/66874 (PMC12680937; doi:10.2196/66874)
Supplement: Multimedia Appendix 3 [file jmir_v27i1e66874_app3.docx]

Appendix C1: Summary of studies

| Author and year | Study design | Target condition | Estimated  Treatment burden level | Nr. of participants | Intervention type | Intervention components |
| --- | --- | --- | --- | --- | --- | --- |
| Eton et al., 2024 [13] | Mixed methods | Multimorbidity | Unsure | 215 | Electronic point-of-care tool | Electronic questionnaire |
| Fairbrother et al., 2014 [45] | Qualitative | Chronic heart failure | Not classified | 23 | Telemonitoring | Daily self-assessment  Remote measurements  Educational content |
| Hilliard et al., 2014 [46] | Mixed methods | Cystic Fibrosis | High | 16 | Variety of phone applications | Informational support  Automated functions  Telehealth |
| Högberg et al., 2013 [47] | Qualitative | Lymphoma, Hodgkin’s Lymphoma, Myeloma and Leukemia | High | 17 | Telehealth | Renewing prescriptions, Changing bookings  Support channel |
| Knowles et al., 2021 [48] | Qualitative | Kidney failure | High | 38 | Electronic patient-reported outcomes | Questionnaires |
| Rodger and O'hara, 2019 [49] | Qualitative | Cystic Fibrosis | High | 18 | Wearable devices and mHealth | Remote monitoring  Goal setting |
| Runz-Jørgensen et al., 2017 [50] | Qualitative | Multimorbidity | Low and High | 10 | eHealth | Remote appointments and monitoring  Self-monitoring  Drug reminders |
| Ryu et al., 2023 [51] | Qualitative | Multimorbidity | Not classified | 23 | Visualization tool | Value elicitation  Facilitated use of the tool |
| Walsh et al., 2019 [52] | Qualitative | Ulcerative colitis | Medium/Low | 66 | Remote monitoring | Questionnaires |
| Pichon et al., 2021 [53] | Qualitative | Endometriosis | Medium/Low | 31 | Any digital intervention | Self-tracking  Reflection tools  Tools facilitating sensemaking |
| Tran et al., 2019 [54] | Mixed methods | Diabetes, asthma, rheumatologic conditions, neurological  disorders, and cancer | Low and High | 1183 | Wearable devices and AI | AI screening  Wearable sensors  AI chatbot |
| van Schelven et al., 2023 [55] | Qualitative | Diabetes, Rheumatism, Cerebral palsy and Scoliosis. | Not classified | 10 | Digital tool for body mapping | Digital body map builder  Chat robot |
| Al Zahidy et al., 2025 [56] | Qualitative | Diabetes | Medium/Low | 20 | Digital medicine tools for diabetes management | Glucose monitors, Insulin pumps, Patient portals, Self management applications |
| Luc et al., 2025 [58] | Qualitative | Chronic neck pain | Medium/Low | 11 | VR serious games |  |
| Polus et al., 2024 [59] | Case study | Epilepsy | Medium/Low | 3 | Digital care pathway | Messaging HCPs, Seizure diary, Questionnaires |
| Shewamene et al., 2024 [60] | Qualitative | Tuberculosis | High | 57 | Digital adherence technologies | Smart pillbox, Medication labels, Audio-visual reminders |
| Skovlund et al., 2023 [61] | Mixed methods | Diabetes | High | 9 | Any digital tools | eHealth  Remote monitoring telehealth |
| Watanabe et al., 2023 [62] | Non-randomised experimental study | Cystic fibrosis | High | 31 | Smartphone app | Activity tracking  Telehealth  Dashboard |
| McBride et al., 2020 [63] | Qualitative | Hypertension | Medium/Low | 11 | Self-management app | Self-monitoring of blood pressure (BP)  Medication reminders Visualisation of BP |
| Betsema et al., 2025 [64] | Qualitative | Cystic fibrosis | High | 11 | Applications and digital monitoring | Medication reminders, Ordering medication, Tracking lifestyle factors, Measuring blood glucose, Psychological support |
| Duckworth et al., 2024 [65] | Mixed methods | COPD | Medium/Low | Qualitative: 15  App user data: 243 | mHealth application | Educational content, pulmonary rehabilitation, Localised weather/pollution levels, Digital diaries |
| Wali et al., 2023 [66] | Qualitative | Pediatric heart disease | Medium/Low | 8 | Digital therapeutic intervention | Real-time data and remote support |
| Morton et al., 2018 [67] | Qualitative | High blood pressure | Medium/Low | 35 | Self-monitoring tool | Self-monitoring of BP  Lifestyle change modules  Medication change |
| Chai et al., 2022 [68] | Mixed methods | Cancer | High | 24 | Mobile game for children | Pet care  Mini-games  Educational module  Alarm  Diary  Incentive module  Help module |
| Fergus et al., 2014 [69] | Mixed methods | Breast cancer | High | 20 | Informational program | Learning modules  Articles |
| Jung et al., 2021 [70] | Cross-sectional | Bladder cancer | High | 630 | Any web-based information | Informational resources  Peer support |
| Robinson et al., 2020 [71] | Non-randomized experimental study | Type 1 Diabetes | High | 4 | Social robot | Informational support Councelling  Mental imagery |
| Stringer et al., 2023 [72] | Qualitative | Head and neck cancer | High | 12 | Decision aid | Informational support  Decision aid |
| Bouamrane et al., 2019 [73] | Qualitative | Kidney failure | High | 9 | Patient portal | Dashboard  QoL tool  Data recording  Care pathway |
| Isika, Mendoza & Bosua,  2020 [74] | Mixed methods | Fibromyalgia | Medium/Low | 10 | Social media (Instagram) | Peer support in social media |
| Raaijmakers et al., 2023 [75] | Mixed methods | Multimorbidity | Not classified | 61 | Self-management tools | Questionnaires  Self-measurements  Training modules |
| Cleres et al., 2021 [76] | Design science | COPD | Medium/Low | 4 | Voice-based conversational agent | Asking symptom-related questions |
| Dhingra et al., 2020 [77] | Cross-sectional | Cystic Fibrosis | High | 74 | Screening-and-triage model | Web-based screening |
| Meiklem et al., 2022 [78] | Mixed methods | Chronic kidney disease | High | 101 | Patient portal | Questionnaires |
| Huuskes et al., 2021 [79] | Qualitative | Kidney transplant recipients | High | 34 | Telehealth | Remote appointments |
| Uchiyama et al., 2022 [80] | Randomized controlled trial | End-stage renal disease | High | 15 | Remote monitoring | Patient data sharing |
| Sabesan et al., 2014 [81] | Qualitative | Cancer | High | 35 | Telehealth | Remote consultations |
| Flythe et al., 2022 [82] | Qualitative | Kidney failure | High | 36 | Wearable dialysis device |  |
| Hale et al., 2023 [83] | RCT | Asthma | Medium/Low | 200 | Adherence recording  device | Remote monitoring |
| Schrader et al., 2014 [84] | Qualitative | Multimorbidity | Not classified | 8 | Online management program | Interview  Goals assessment |
| Doyle et al., 2017 [85] | Qualitative | Multimorbidity | Not classified | 124 | Medication management application | Medication list  Information of medications  Support for medication taking |
| Doyle et al., 2019 [86] | Qualitative | Multimorbidity | Not classified | 125 (overlapping with Doyle et al., 2017) | Design recommendations for digital interventions | Daily checklists  Triage services  Informational support |
| Ongwere et al., 2018 [87] | Qualitative | Multimorbidity with type-2 diabetes | Not classified | 15 | Design suggestions for digital interventions |  |
| Spencer-Bonilla et al., 2021 [88] | Mixed methods | Type 2 Diabetes | Medium/Low | 160 | Smartphone application | Questionnaires  Self-management tasks |
| Meiklem et al., 2021 [89] | Case study | Chronic kidney disease | High | 33 | Patient portal | Questionnaires |
| Wildman et al., 2022 [90] | RCT | Cystic fibrosis | High | 607 | eHealth | Data-logging nebulisers Digital platform,  Behavioural change  sessions with trained clinical interventionists |

Appendix C2: Examples of the conversion of quantitative data to qualitative

| Author and year | Original quantitative data | Converted qualitative data |
| --- | --- | --- |
| Watanabe et al., 2023 [62] | Most patients (67%) said the app improved confidence in and motivation for continuing their regimen. | The intervention increased motivation and confidence for treatment regimen. |
| Uchiyama et al., 2022 [80] | Significant improvements were observed in the TSQM-9 subscale of Convenience (76.3±15.4 vs. 63.3±17.3; P<0.001). Intervention reduced consultation time during regular monthly visits (813±269 vs. 1024±292 s; P<0.001). | The intervention reduced treatment burden by increasing convenience and reducing time spent on consultation visits. |
| Wildman et al., 2022 [90] | Seven CFQ-R subscales showed treatment burden reduced for the intervention (3.9 (1.2 to 6.7) points). | Intervention reduced treatment burden relevant to Cystic Fibrosis |

Appendix C3: Illustrative examples of coding

| Author and year | Quote of coded text | Codes |
| --- | --- | --- |
| Al Zahidy et al., 2025 [56] | Under personal factors, individuals re-  ported the impact of perceived accuracy and  trust in the devices and of inaccurate readings  on treatment decisions. This often stemmed  from previous experiences with devices that  were inaccurate, leading to diminished trust  and making dependence on technology more  challenging. | Concerns of accuracy |
| McBride et al., 2020 [63] | Feeling empowered, some reported instances during which theapp had encouraged them to play an active role in consultations.This occurred when patients used the app to guide conversationwith their general practitioner (GP). | Empowerment |
| Morton et al., 2018 [67] | Some participants were motivated to increase their physical activity, engage in stress management activities or healthy eating because they could see this had a positive impact on their BP readings. This helped them feel more in control of their BP. | Supporting self-management, Increased motivation for treatment |
| Chai et al., 2022[68] | The results showed that overall, the children’s intention to undergo cancer treatment had increased significantly | Increased motivation for treatment |
| Isika et al., [74] | Among a range of influences, information support  exchanges to better manage living with fibromyalgia | Providing informational support |
| Dhingra et al., 2020 [77] | The operational elements of the model comprised repeated web-based screening, triggered evaluation, and triage to CF professionals  for early intervention. | Facilitated tools |
| Huuskes et al., 2021[79] | Some participants found that at times “technology hasn’t stood up” which delayed appointments resulting in telephone calls instead of video calls. | Technical problems |
| Huuskes et al., 2021[79] | Telehealth is the use of telecommunication, usuallyby telephone or video call, to provide a clinical consul-tation | Telehealth |

References:

[13] D. Eton *et al.*, “Building a measurement framework of burden of treatment in complex patients with chronic conditions: a qualitative study,” *Patient Relat Outcome Meas*, p. 39, Aug. 2012, doi: 10.2147/PROM.S34681.

[45] P. Fairbrother *et al.*, “Telemonitoring for chronic heart failure: the views of patients and healthcare professionals – a qualitative study,” *J Clin Nurs*, vol. 23, no. 1–2, pp. 132–144, 2014, doi: <https://doi.org/10.1111/jocn.12137>.

[46] M. E. Hilliard, A. Hahn, A. K. Ridge, M. N. Eakin, and K. A. Riekert, “User Preferences and Design Recommendations for an mHealth App to Promote Cystic Fibrosis Self-Management,” *JMIR mHealth uHealth*, vol. 2, no. 4, pp. e44-, 2014, doi: 10.2196/mhealth.3599.

[47] K. Högberg, L. Sandman, M. Nyström, D. Stockelberg, and A. Broström, “Prerequisites required for the provision and use of web-based communication for psychosocial support in haematologic care,” *European Journal of Oncology Nursing*, vol. 17, no. 5, pp. 596–602, 2013, doi: <https://doi.org/10.1016/j.ejon.2013.01.005>.

[48] S. E. Knowles, A. Ercia, F. Caskey, M. Rees, K. Farrington, and S. N. Van der Veer, “Participatory co-design and normalisation process theory with staff and patients to implement digital ways of working into routine care: the example of electronic patient-reported outcomes in UK renal services.,” *BMC Health Serv Res*, vol. 21, no. 1, pp. 1–11, 2021, doi: 10.1186/s12913-021-06702-y.

[49] S. Rodger and K. O’Hara, “Exploring the Potential for Technology to Improve Cystic Fibrosis Care Provision: Patient and Professional Perspectives,” *Proc. ACM Hum.-Comput. Interact.*, vol. 3, no. CSCW, p., 2019, doi: 10.1145/3359223.

[50] S. M. Runz-Jørgensen, M. L. Schiøtz, and U. Christensen, “Perceived value of eHealth among people living with multimorbidity: a qualitative study.,” *J Comorb*, vol. 7, no. 1, pp. 96–111, 2017, doi: 10.15256/joc.2017.7.98.

[51] H. Ryu *et al.*, “‘You Can See the Connections’: Facilitating Visualization of Care Priorities in People Living with Multiple Chronic Health Conditions,” in *Proceedings of the 2023 CHI Conference on Human Factors in Computing Systems*, New York, NY, USA: Association for Computing Machinery, 2023, p. doi: 10.1145/3544548.3580908.

[52] A. *et al.*, “Real-time data monitoring for ulcerative colitis: patient perception and qualitative analysis.,” *Intest Res*, vol. 17, no. 3, pp. 365–374, 2019, doi: 10.5217/ir.2018.00173.

[53] A. Pichon *et al.*, “Divided We Stand: The Collaborative Work of Patients and Providers in an Enigmatic Chronic Disease,” *Proc. ACM Hum.-Comput. Interact.*, vol. 4, no. CSCW3, p., 2021, doi: 10.1145/3434170.

[54] V. T. Tran, C. Riveros, and P. Ravaud, “Patients’ views of wearable devices and AI in healthcare: findings from the ComPaRe e-cohort.,” *NPJ Digit Med*, vol. 2, p. 53, 2019, doi: 10.1038/s41746-019-0132-y.

[55] van S. F, van der M. E, E. Wessels, and H. R. Boeije, “Let Us Talk Treatment: Using a Digital Body Map Tool to Examine Treatment Burden and Coping Strategies Among Young People with a Chronic Condition.,” *Patient Prefer Adherence*, vol. 17, pp. 517–529, 2023, doi: 10.2147/PPA.S400702.

[56] M. A. Al Zahidy *et al.*, “Digital Medicine Tools and the Work of Being a Patient: A Qualitative Investigation of Digital Treatment Burden in Patients With Diabetes,” *Mayo Clinic Proceedings: Digital Health*, vol. 3, no. 1, p. 100180, Mar. 2025, doi: 10.1016/j.mcpdig.2024.11.001.

[58] A. Luc, N. Lambricht, I. Aujoulat, C. Detrembleur, and L. Pitance, “Experiences of People With Persistent Nonspecific Neck Pain Who Used Immersive Virtual Reality Serious Games in the Home Setting: A Qualitative Study,” *Phys Ther*, vol. 105, no. 3, Mar. 2025, doi: 10.1093/ptj/pzae149.

[59] M. Polus *et al.*, “The Role of Digital Care Pathway for Epilepsy on Patients’ Treatment Burden: Clinicians’ Perspective,” 2024, pp. 257–268. doi: 10.1007/978-3-031-59080-1_19.

[60] Z. Shewamene *et al.*, “Facilitators and barriers to uptake of digital adherence technologies in improving TB care in Ethiopia: A qualitative study,” *PLOS Digital Health*, vol. 3, no. 11, p. e0000667, Nov. 2024, doi: 10.1371/journal.pdig.0000667.

[61] S. E. Skovlund, S. Renza, J. Laurent, and P. Cerletti, “Identification of Core Outcome Domains and Design of a Survey Questionnaire to Evaluate Impacts of Digital Health Solutions That Matter to People With Diabetes.,” *J Diabetes Sci Technol*, pp. 19322968231179740-, 2023, doi: 10.1177/19322968231179740.

[62] A. H. Watanabe *et al.*, “Patient Perspectives on the Use of Digital Technology to Help Manage Cystic Fibrosis.,” *Pulm Med*, vol. 2023, p. 5082499, 2023, doi: 10.1155/2023/5082499.

[63] C. M. McBride, E. C. Morrissey, and G. J. Molloy, “Patients’ Experiences of Using Smartphone Apps to Support Self-Management and Improve Medication Adherence in Hypertension: Qualitative Study.,” *JMIR Mhealth Uhealth*, vol. 8, no. 10, pp. e17470-, 2020, doi: 10.2196/17470.

[64] L. Betsema, M. Yang, A. Bohr, A. Herrera, and S. Kaae, “Cystic fibrosis patients’ preferences for electronic devices that monitor their inhalation – A qualitative study,” *Respir Med*, vol. 238, p. 107980, Mar. 2025, doi: 10.1016/j.rmed.2025.107980.

[65] C. Duckworth *et al.*, “Characterising user engagement with mHealth for chronic disease self-management and impact on machine learning performance,” *NPJ Digit Med*, vol. 7, no. 1, p. 66, Mar. 2024, doi: 10.1038/s41746-024-01063-2.

[66] S. Wali, A. Remtulla Tharani, D. Balmer-Minnes, J. A. Cafazzo, J. Laks, and A. Jeewa, “Exploring the use of a digital therapeutic intervention to support the pediatric cardiac care journey: Qualitative study on clinician perspectives,” *PLOS Digital Health*, vol. 2, no. 12, p. e0000371, Dec. 2023, doi: 10.1371/journal.pdig.0000371.

[67] K. Morton *et al.*, “Qualitative process study to explore the perceived burdens and benefits of a digital intervention for self-managing high blood pressure in Primary Care in the UK,” *BMJ Open*, vol. 8, no. 5, p., 2018, doi: 10.1136/bmjopen-2017-020843.

[68] C. W. E. Chai, B. T. Lau, M. K. T. Tee, and A. Al Mahmud, “Evaluating a serious game to improve childhood cancer patients’ treatment adherence,” *Digit Health*, vol. 8, p., 2022, doi: 10.1177/20552076221134457.

[69] K. D. Fergus *et al.*, “Development and pilot testing of an online intervention to support young couples’ coping and adjustment to breast cancer,” *Eur J Cancer Care (Engl)*, vol. 23, no. 4, pp. 481–492, 2014, doi: <https://doi.org/10.1111/ecc.12162>.

[70] A. Jung *et al.*, “Well-being and Perceptions of Supportive Resources among Caregivers of Patients with Bladder Cancer,” *Bladder Cancer*, vol. 7, no. 1, pp. 43–52, 2021, doi: 10.3233/BLC-200412.

[71] N. L. Robinson, J. Connolly, L. Hides, and D. J. Kavanagh, “A Social Robot to Deliver an 8-Week Intervention for Diabetes Management: Initial Test of Feasibility in a Hospital Clinic,” in *Social Robotics: 12th International Conference, ICSR 2020, Golden, CO, USA, November 14–18, 2020, Proceedings*, Berlin, Heidelberg: Springer-Verlag, 2020, pp. 628–639. doi: 10.1007/978-3-030-62056-1_52.

[72] E. Stringer, J. J. Lum, J. Livergant, and A. W. Kushniruk, “Decision Aids for Patients With Head and Neck Cancer: Qualitative Elicitation of Design Recommendations From Patient End Users,” *JMIR Hum Factors*, vol. 10, p., 2023, doi: 10.2196/43551.

[73] M.-M. Bouamrane *et al.*, “Haemodialysis electronic patient portal: A design requirements analysis and feasibility study with domain experts,” in *Proceedings - IEEE Symposium on Computer-Based Medical Systems*, 2019, pp. 212–216. doi: 10.1109/CBMS.2019.00051.

[74] N. Isika, A. Mendoza, and R. Bosua, “‘I Need to Compartmentalize Myself’: Appropriation of Instagram for Chronic Illness Management,” in *Proceedings of the Australasian Computer Science Week Multiconference*, New York, NY, USA: Association for Computing Machinery, 2020, p. doi: 10.1145/3373017.3373040.

[75] L. Raaijmakers, J. Vercoulen, T. Schermer, and E. Bischoff, “OPTIMA FORMA - Towards a patient-centred multimorbidity approach for chronic disease management in primary care...22nd International Conference on Integrated Care, May 23-25, 2022, Odense, Denmark.,” *International Journal of Integrated Care (IJIC)*, vol. 22, pp. 1–2, doi: 10.5334/ijic.ICIC22014.

[76] D. Cleres, F. Rassouli, M. Brutsche, T. Kowatsch, and F. Barata, “Lena: A Voice-Based Conversational Agent for Remote Patient Monitoring in Chronic Obstructive Pulmonary Disease,” in *CEUR Workshop Proceedings*, 2021, p. [Online]. Available: https://www.scopus.com/inward/record.uri?eid=2-s2.0-85110528552&partnerID=40&md5=c89b4cda5b6594a6adf6da4ba1eba898

[77] L. Dhingra *et al.*, “Addressing the burden of illness in adults with cystic fibrosis with screening and triage: An early intervention model of palliative care.,” *J Cyst Fibros*, vol. 19, no. 2, pp. 262–270, 2020, doi: 10.1016/j.jcf.2019.08.009.

[78] R. Meiklem *et al.*, “Patients’ and Clinicians’ Perspectives on the Acceptability of Completing Digital Quality of Life Questionnaires During Routine Haemodialysis Clinics: A Mixed-Methods Study,” in *Studies in Health Technology and Informatics*, 2022, pp. 752–756. doi: 10.3233/SHTI220179.

[79] B. M. Huuskes *et al.*, “Kidney transplant recipient perspectives on telehealth during the COVID-19 pandemic.,” *Transpl Int*, vol. 34, no. 8, pp. 1517–1529, 2021, doi: 10.1111/tri.13934.

[80] K. Uchiyama *et al.*, “Effects of a remote patient monitoring system for patients on automated peritoneal dialysis: a randomized crossover controlled trial.,” *Int Urol Nephrol*, vol. 54, no. 10, pp. 2673–2681, 2022, doi: 10.1007/s11255-022-03178-5.

[81] S. Sabesan, J. Kelly, R. Evans, and S. Larkins, “A tele-oncology model replacing face-to-face specialist cancer care: perspectives of patients in North Queensland,” *J Telemed Telecare*, vol. 20, no. 4, pp. 207–211, 2014, doi: 10.1177/1357633X14529237.

[82] J. E. Flythe *et al.*, “Development of a Patient Preference Survey for Wearable Kidney Replacement Therapy Devices.,” *Kidney360*, vol. 3, no. 7, pp. 1197–1209, 2022, doi: 10.34067/KID.0001862022.

[83] E. M. Hale *et al.*, “Use of digital measurement of medication adherence and lung function to guide the management of uncontrolled asthma (INCA Sun): a multicentre, single-blinded, randomised clinical trial.,” *Lancet Respir Med*, vol. 11, no. 7, pp. 591–601, 2023, doi: 10.1016/S2213-2600(22)00534-3.

[84] G. Schrader *et al.*, “An eHealth Intervention for Patients in Rural Areas: Preliminary Findings From a Pilot Feasibility Study.,” *JMIR Res Protoc*, vol. 3, no. 2, pp. e27-, 2014, doi: 10.2196/resprot.2861.

[85] J. Doyle *et al.*, “Addressing Medication Management for Older People with Multimorbidities: A Multi-Stakeholder Approach,” in *Proceedings of the 11th EAI International Conference on Pervasive Computing Technologies for Healthcare*, New York, NY, USA: Association for Computing Machinery, 2017, pp. 78–87. doi: 10.1145/3154862.3154883.

[86] J. Doyle *et al.*, “Managing Multimorbidity: Identifying Design Requirements for a Digital Self-Management Tool to Support Older Adults with Multiple Chronic Conditions,” in *Proceedings of the 2019 CHI Conference on Human Factors in Computing Systems*, New York, NY, USA: Association for Computing Machinery, 2019, pp. 1–14. doi: 10.1145/3290605.3300629.

[87] T. Ongwere, G. Cantor, S. R. Martin, P. C. Shih, J. Clawson, and K. Connelly, “Design Hotspots for Care of Discordant Chronic Comorbidities: Patients’ Perspectives,” in *Proceedings of the 10th Nordic Conference on Human-Computer Interaction*, New York, NY, USA: Association for Computing Machinery, 2018, pp. 571–583. doi: 10.1145/3240167.3240221.

[88] G. Spencer-Bonilla *et al.*, “Patient Work and Treatment Burden in Type 2 Diabetes: A Mixed-Methods Study.,” *Mayo Clin Proc Innov Qual Outcomes*, vol. 5, no. 2, pp. 359–367, 2021, doi: 10.1016/j.mayocpiqo.2021.01.006.

[89] R. Meiklem *et al.*, “Advanced Kidney Disease Patient Portal: Implementation and Evaluation with Haemodialysis Patients,” in *Lecture Notes in Computer Science (including subseries Lecture Notes in Artificial Intelligence and Lecture Notes in Bioinformatics)*, 2021, pp. 175–196. doi: 10.1007/978-3-030-85616-8_12.

[90] M. J. Wildman *et al.*, “Self-management intervention to reduce pulmonary exacerbations by supporting treatment adherence in adults with cystic fibrosis: a randomised controlled trial.,” *Thorax*, vol. 77, no. 5, pp. 461–469, 2022, doi: 10.1136/thoraxjnl-2021-217594.
